# Ventricular outflow tract obstruction: An *in-silico* model to relate the obstruction to hemodynamic quantities in cardiac paediatric patients

**DOI:** 10.1371/journal.pone.0258225

**Published:** 2021-10-15

**Authors:** Giulia Comunale, Massimo Padalino, Carmelo Maiorana, Giovanni Di Salvo, Francesca M. Susin

**Affiliations:** 1 Cardiovascular Fluid Dynamics Laboratory HER, Department of Civil, Environmental and Architectural Engineering–University of Padova, Padova, Italy; 2 Paediatric and Congenital Cardiovascular Surgery Unit, Department of Cardio-thoracic and Vascular Sciences and Public Health, University of Padova Medical School, Padova, Italy; 3 Department of Civil, Environmental and Architectural Engineering–University of Padova, Padova, Italy; 4 Paediatric Cardiology, Department of Woman and Child’s Health, University of Padova Medical School, Padova, Italy; Policlinico S. Orsola-Malpighi, ITALY

## Abstract

**Background:**

Right (R) or left (L) ventricular outflow tract (VOT) obstruction can be either a dynamic phenomenon or a congenital anatomic lesion, which requires a prompt and optimal timing of treatment to avoid a pathological ventricular remodelling.

**Objective:**

To develop a simple and reliable numerical tool able to relate the R/L obstruction size with the pressure gradient and the cardiac output. To provide indication of the obstruction severity and be of help in the clinical management of patients and designing the surgical treatment for obstruction mitigation.

**Methods:**

Blood flow across the obstruction is described according to the classical theory of one-dimensional flow, with the obstruction uniquely characterized by its size. Hemodynamics of complete circulation is simulated according to the lumped parameter approach. The case of a 2 years-old baby is reproduced, with the occlusion placed in either the R/ or the L/VOT. Conditions from wide open to almost complete obstruction are reproduced.

**Results:**

Both R/LVOT obstruction in the *in-silico* model resulted in an increased pressure gradient and a decreased cardiac output, proportional to the severity of the VOT obstruction and dependent on the R/L location of the obstruction itself, as it is clinically observed.

**Conclusion:**

The *in-silico* model of ventricular obstruction which simulates pressure gradient and/or cardiac output agrees with clinical data, and is a first step towards the creation of a tool that can support the clinical management of patients from diagnosis to surgical treatments.

## Introduction

Obstruction of the right (R) or left (L) ventricular outflow tract (VOT) is a common pathological cardiac disease which is characterized by a partial or a complete VOT obstruction that causes several hemodynamic and pathological changes. This pathology may be congenital or acquired, and may cause ventricular dysfunction and symptoms at any age. When congenital, VOT obstruction may be involving the right or left ventricular outflow tract with a spectrum of disorders. For example, in the paediatric population, a right ventricular outflow tract obstruction (RVOTO) may be due to a defect in the pulmonic valve, the supravalvar region, the infundibulum, or the pulmonary artery, and include pulmonary atresia or stenosis, and tetralogy of Fallot [1]. Analogously, a left ventricular outflow tract obstruction (LVOTO) may be due to a defect in the aortic valve, or a defect located at the subvalvar or supravalvar level, and include aortic valve stenosis, coarctation of the aorta and hypoplastic left heart syndrome [2]. Moreover, R/LVOTO may also be caused by primary cardiac tumours in the proximity of the pulmonary or aortic valve. These cardiac tumours arise in the heart, and, usually, they are characterized by round or oval shape with a smooth or lobulated surface [3].

Despite the clinical relevance of VOT obstruction (e.g., aortic valve stenosis alone, which accounts for 3% to 6% of congenital heart disease (CHD), has an incidence of 2.5–6 per 10000 live births [2]), several lacunae still exist.

*i)* From the clinical point of view, the accurate non-invasive prediction of VOT severity for appropriate timing of intervention is still challenging [4]. The indications for treatment of R/LVOT obstruction, reported by the American Heart Association [5], refer to the peak-to-peak ventriculo-arterial gradient derived by cardiac catheterization in sedated patients. This value is consistently lower than what obtained by echocardiography, which is the most common measurement used to assess disease severity in clinical practice [2]. This is particularly significant in those patients with small aortic or pulmonary arterial diameters, e.g., paediatric population [4, 6]. Moreover, notice also that how the obstruction geometry affects the pressure gradient is one further open question [4]. For these reasons, the obstruction evaluation by echocardiography may be unsuitable due to overestimation of the pressure gradient, resulting in misclassification of the disease severity and leading to inappropriate timing of intervention.

*ii)* To the best of our knowledge, currently engineering research has focused its attention almost exclusively to aortic valve stenosis [7–9]. The physical and mathematical-numerical models that have been proposed over the years to describe aortic transvalvular hemodynamics are numerous, and with varying degrees of detail e.g., from bulk flow parameters to local flow kinematic and dynamics, from rigid valve geometry to time dependent geometry, calcification modelling, and so on [8–12]. Modelling of obstructive diseases other than aortic stenosis has been only sporadic and limited to the adult population. Thus, developing a mathematical model that can relate the percentage of VOT diameter reduction (i.e., obstruction) to the pressure gradient and the cardiac output may help to overcome the stated drawbacks with the final goal to optimize diagnosis of an obstructive disease, and clinical management.

In this study, we aimed to derive a numerical model that can reproduce the hemodynamic changes occurring with R/LVOT obstruction, and to relate the percentage of restriction to overall hemodynamic quantities. A lumped-parameter model of the complete circulation is hence coupled to a local model of hemodynamics through the narrowing, being fixed the degree of obstruction. As a result, the model gives both the cardiac output and the pressure gradient through the narrow tract. We applied the model to a paediatric case since VOT obstruction in infants may become hemodynamically significant earlier than in adults, due to the rapid rate of growth and the small cardiac dimensions [13], and echocardiographic assessment may be misled. Nevertheless, note that the model can be easily adapted to the adult case by appropriately tuning the model’s parameters.

## Methods

### *In-silico* model

From the fluid dynamic point of view any cardiovascular obstruction, located in either vessels or heart chambers, can be seen as an obstacle to blood flow and, as such, as a source of disturbances that subtract mechanical energy to the bulk flow in the form of an irreversible pressure loss. When the bulk flow develops along one main direction, as in the case of vessels or, for the heart chambers, in the left or right ventricular outflow tract, seen as a cylindrical conduit [14, 15], the behaviour of the obstructed blood flow can be effectively described by means of the classical equations of one-dimensional hydrodynamics [16]. Such an approach, which allows for the evaluation of global hemodynamic parameters, has been widely adopted to investigate cardiovascular flows in either physiological or pathological conditions. For example, a large amount of work has been done in the past twenty years to develop mathematical models able to reliably predict the pressure gradient across a stenotic aortic valve [7, 9].

**Fig 1:**
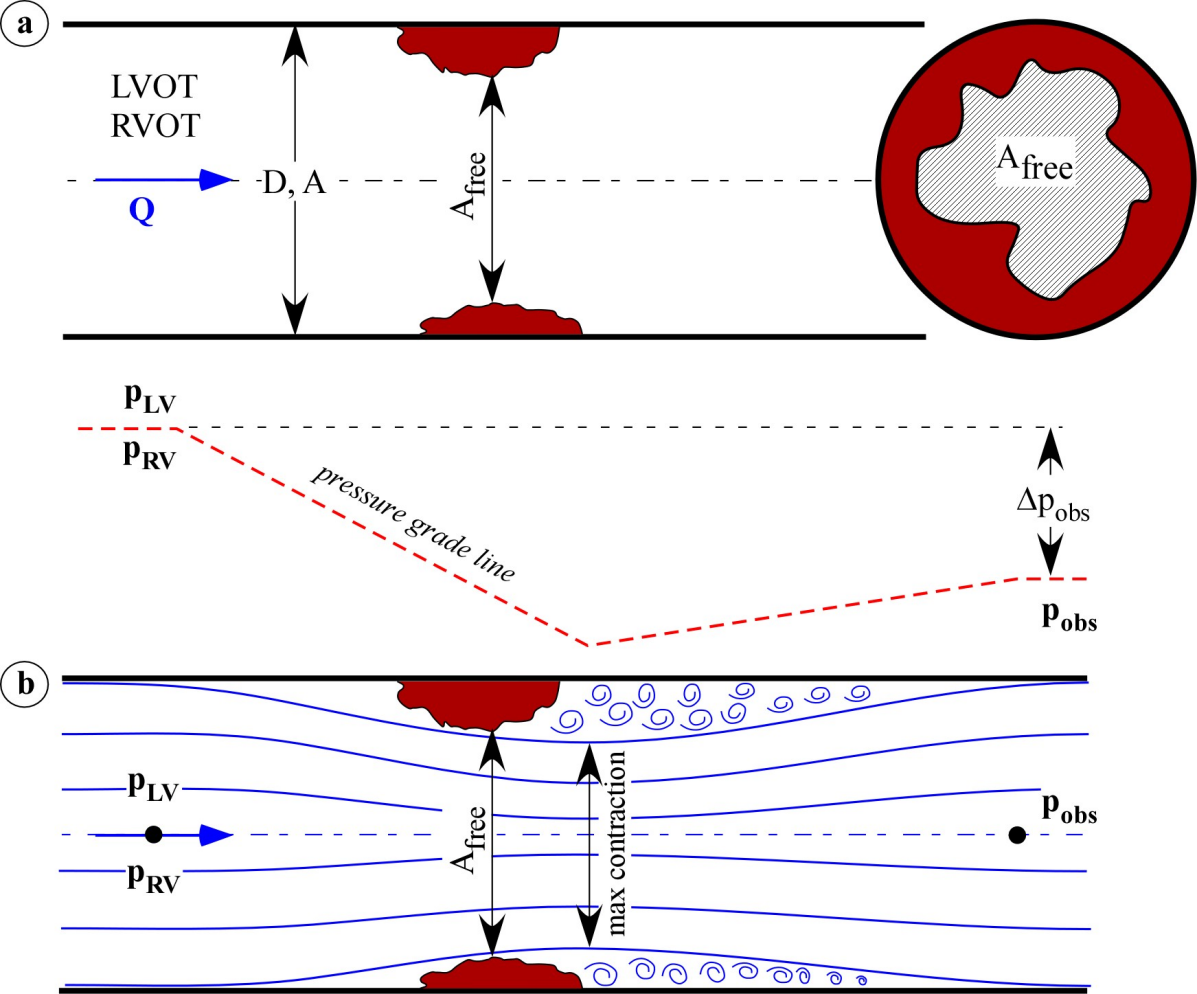
(a) Scheme of the ventricular outflow tract simulated as a one-dimensional flow in a circular pipe of diameter D and area A. The obstruction reduces the cross-sectional area to the minimum A_free_. (b) Streamlines of the flow and the pressure grade line (red dotted line) due to the presence of the obstruction. The obstruction causes a pressure drop which is only partially recovered in the downstream section, determining a pressure gradient Δ*p*_*obs*_. Eddies are present only on the downward section toward the artery. Q, the flow rate, p_LV_ (p_RV_), pressure in the left (right) ventricle, and p_obs_, pressure distal to the obstruction.

**Fig 2:**
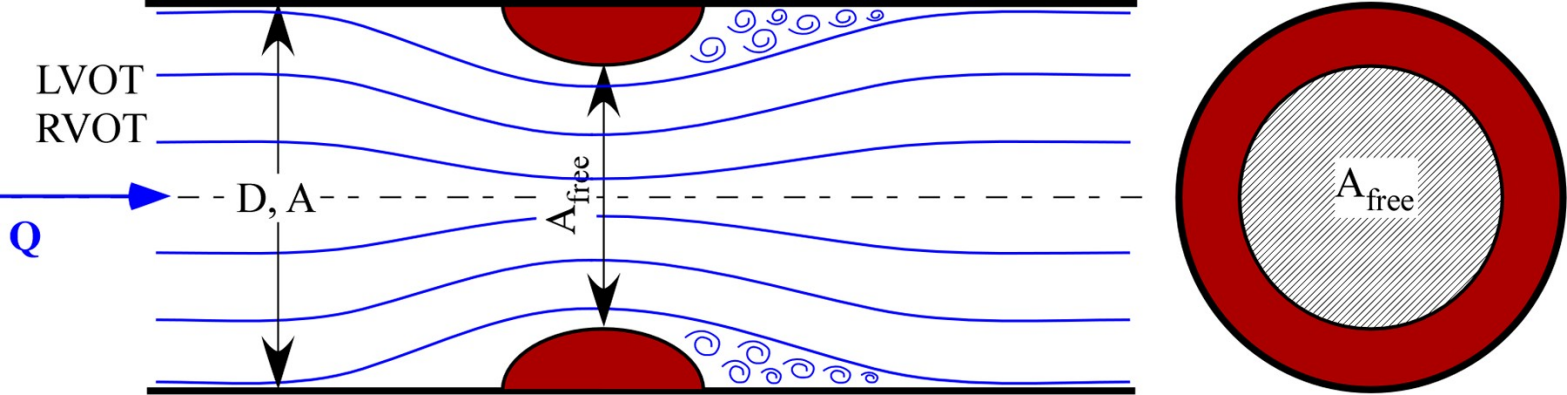
Streamlines of the flow due to the presence of an ideal round obstruction.

Here, following that same approach, the blood flow through an obstruction located in either R/LVOT (such as a localized tumour mass, a subvalvular stenosis, or a supravalvular stenosis) is assimilated to the one-dimensional flow in a circular pipe of diameter *D* and area *A*, partly occluded by the obstruction (Fig 1A). The latter is geometrically described by the minimum cross-sectional area that the obstacle locally leaves free to the bulk flow, *A*_*free*_, and is considered of negligible length [14]. Due to the narrowing of the pipe, the flow is not able to maintain its proximal undisturbed character but rather behaves as depicted in Fig 1B i.e., it initially contracts up to the minimum area and then expands exhibiting distal vortices that cause irreversible viscous energy dissipation. As a consequence, blood velocity increases (decreases) due to the flow area contraction (expansion) and blood pressure does the opposite. However, viscous losses impede the pressure to fully recover so that the pressure gradient Δ*p*_*obs*_ between the left (right) ventricle and the section distal to the obstruction develops. The gradient can hence be calculated as (see S1 File for details)

$$\Delta p_{obs}=K_{obs}∙Q^{2}$$ (1)

where *Q* is the (instantaneous) flow rate through the obstruction (i.e., the flow rate ejected by the left or the right ventricle, depending on the obstruction location) and *K*_*obs*_ is a coefficient that depends on the shape and size of the obstacle. Following the approach reported in [7], the latter is here assumed as

$$K_{obs}=f_{shape}\frac{\rho }{2}{\left(\frac{1}{A_{free}}-\frac{1}{A}\right)}^{2}$$ (2)

with *ρ* the blood density and *f*_*shape*_ a factor ≥1 that accounts for the obstruction morphology. Notice that *f*_*shape*_ = 1 corresponds to the case of an ideal round, annular, concentric obstacle (Fig 2). Notice also that Eq (2) assumes negligible friction losses along the stenotic lesion i.e., a localized constriction mainly subjected to mechanical energy losses due to wall separation phenomena is considered [14].

Eqs (1) and (2) represent the mathematical model of the flow in the narrowing, and allow the calculation of the pressure gradient across the obstruction Δ*p*_*obs*_ given the narrowing ratio *A*_*free*_/*A* and the ejected flow rate *Q*. However, it is worth stressing that the presence of the obstruction in the R/LVOT may induce progressive ventricular dysfunction due to prolonged increased afterload, and ultimately reduce ventricular stroke volume and hence the flow rate *Q* and the cardiac output, CO. For that reason, and in order to compute not only Δ*p*_*obs*_ but also *Q* and CO as a function of *A*_*free*_/*A*, Eqs (1) and (2) are coupled to a previously developed lumped parameter model (0D) of the complete cardiovascular circulation [17]. The 0D model reduces the cardiovascular system to the sum of multiple functional compartments (e.g., atria and ventricles, large vessels, microvasculature), and describes the hydrodynamic functionality of each compartment by suitably relating the local instantaneous pressure and blood flow rate/volume. Parameters peculiar to each compartment (e.g., heart chambers’ elastance, large vessels’ compliance, microvasculature’s resistance) are adopted to characterize the compartment functionality and account for its effect on the complete circulation. As a result, the instantaneous pressure and flow rate are obtained in each compartment from the 0D model. A complete description of the model itself is given in S3 File. Here, we just recall that the model describes the flow across the heart valves by a relationship of the kind Δ*p*_*v*_ = *R*_*v*_∙*Q*, where Δ*p*_*v*_ is the transvalvular pressure drop and *R*_*v*_ is the valve resistance parameter. For the aortic and pulmonary valve case, in particular, values for *R*_*v*_ are assumed such that normo-functioning valves are simulated i.e., the pressure gradient across the valve results to be negligible (mean value of Δ*p*_*v*_ around 1–2 mmHg) and does not affect the pressure gradient due to the obstruction located in the ventricular outflow tract.

### Simulations

Conditions pertaining to preserved cardiovascular functionality were reproduced despite the presence of the obstruction i.e., no compensatory mechanism was accounted for even in the most severe obstruction condition. A child case of about 2-years-old with a heart rate HR = 105 bpm, body surface area BSA = 0.55 m^2^, body mass BM = 12 Kg, and left (right) ventricular outflow tract diameter *D* = 0.9 cm (*D* = 1.07 cm) was considered [18]. Physiological values for that case were assigned to the functional parameters contained in the 0D model (see S4 File).

The 0D model alone was first implemented (i.e., no obstruction in either the left or the right ventricular outflow tract was considered) to compute healthy hemodynamics and compare results with clinical data for model validation. Then, the 0D model coupled with Eq (1) and (2) was repeatedly run to reproduce the cardiovascular circulation in presence of the obstruction. Both the cases of obstruction located in the left and in the right ventricular outflow tract were investigated. Simulations were performed for various degrees of obstruction severity by varying the narrowing ratio *A*_*free*_/*A* in the range 0.05–1, 1 being the case of VOT free from obstruction and 0.05 the case of VOT almost completely occluded. Lower *A*_*free*_/*A* ratios were not considered because a further reduction of the flow area determines non-realistic pathological conditions (~ null cardiac output). The shape factor *f*_*shape*_ contained in Eq (2) was assumed equal to 1.

Results computed for each obstructed condition were also post-processed to obtain relevant hemodynamic parameters according to the following relationships

$$CO=\frac{1}{T}\int_{T}Qdt$$ (3)

*T* being the period of one heartbeat;

$$\Delta p_{obs,mean}=\frac{1}{T_{e}}\int_{T_{e}}\Delta p_{obs}dt$$ (4)

where Δ*p*_*obs*,*mean*_ is the mean pressure gradient across the obstacle, calculated in the ejection period *T*_*e*_. The peak value of the gradient, Δ*p*_*obs*,*peak*_, was extracted as the maximum gradient calculated in one heartbeat.

It has now to be noticed that the pressure gradient routinely estimated by Doppler echocardiography in the clinical non-invasive assessment of a stenotic lesion is not Δ*p*_*obs*_. Rather, it is the gradient that establishes between the ventricle and the location of the minimum flow area due to flow acceleration only [9]. The clinical gradient Δ*p*_*Doppler*_ hence does not include the pressure recovery (PR) effect which partially restores the pressure distal to the obstruction [4, 6] and, as a consequence, it is typically larger than Δ*p*_*obs*_. In order to compare Δ*p*_*obs*_ and Δ*p*_*Doppler*_, we hence computed also the latter, by applying the relationship adopted in the clinical practice i.e., the so-called simplified Bernoulli equation

$$\Delta p_{Doppler}=4\cdot V_{A_{free}}^{2}$$ (5)

where $V_{A_{free}}$ is the velocity in the section of minimum flow area, $V_{A_{free}}=Q/A_{free}$. The mean and the peak values of Δ*p*_*Doppler*_ were finally obtained as they were for Δ*p*_*obs*_.

A sensitivity analysis of model results to the shape factor *f*_*shape*_ was performed, which was varied in a realistic range estimated as explained in S2 File.

Finally, a sensitivity analysis to verify the influence of parameters on the model was carried out and reported in S5 File.

The model was run harnessing the built-in MATLAB® function *ode15s*, solving a closed-loop system. The results were obtained after reaching the periodic steady state and they were validated considering literature data.

## Results and discussion

In the clinical setting, a RVOT or LVOT obstruction is a common either congenital or acquired problem. Its clinical management may be compromised by the adoption of the simplified Bernoulli equation used for echocardiography, which does not account for the pressure recovery effect [4, 6]. Beyond that, engineering research has focused almost exclusively on aortic stenosis [7–9], neglecting the wide spectrum of diseases that may cause VOT obstruction. Thus, we developed a mathematical model to correlate the degree of R/LVOT obstruction to the Δ*p* and the cardiac output. The obstructed flow model is very simple, mainly because the lack of detailed clinical data relating the size/morphology of the obstruction and the hemodynamic parameters, in particular in the paediatric population, would make premature any more refined representation. However, real obstructions similar to the simplified one here adopted are not unusual, as for the case of tumour masses in children [3]. Also the lumped model built up for the entire circulation is quite simple, so that parameters can be adequately calibrated also for those patients’ cohorts less represented in the literature i.e., when the ‘reference patient’ is not the average adult male.

Simulations of the healthy condition performed to validate the model compare favourably with literature data, proving a good representation of the reproduced paediatric population: Table 1 reports a quantitative comparison between the output of the healthy child 0D model and literature data for the paediatric population [18]. As it can be seen, global hemodynamic parameters fall within physiological ranges for both the left and right circulation, with differences within the measurement error.

**Table 1:** Comparison of model outputs with literature data specific for the paediatric case given as physiological ranges [18].

Parameters	Unit	Model Output	Reference
SVR	mmHg*s/mL	2.0	1.5–2.1
SBP	mmHg	93	90–110
DBP	mmHg	64	65–75
p_RV_	mmHg	18/2**	15-23/3-7
p_PuA_	mmHg	17/9**	15-23/10-16
p_LA_	mmHg	5.7*	5–10
p_LV_	mmHg	94/3**	90-110/7-9
p_RA_/CVP	mmHg	3.9*	2–6
CO	L/min	2.2	1.3–2.7

HR, heart rate, SVR, systemic vascular resistance, SBP, systolic blood pressure, DBP, diastolic blood pressure, p_RA_, right atrial pressure, p_RV_, right ventricular pressure, p_PuA_, pulmonary artery pressure, p_LA_, left atrial pressure, p_LV_, left ventricular pressure, CVP, central venous pressure, CO, cardiac output.

* mean value

** systolic/diastolic values.

**Fig 3:**
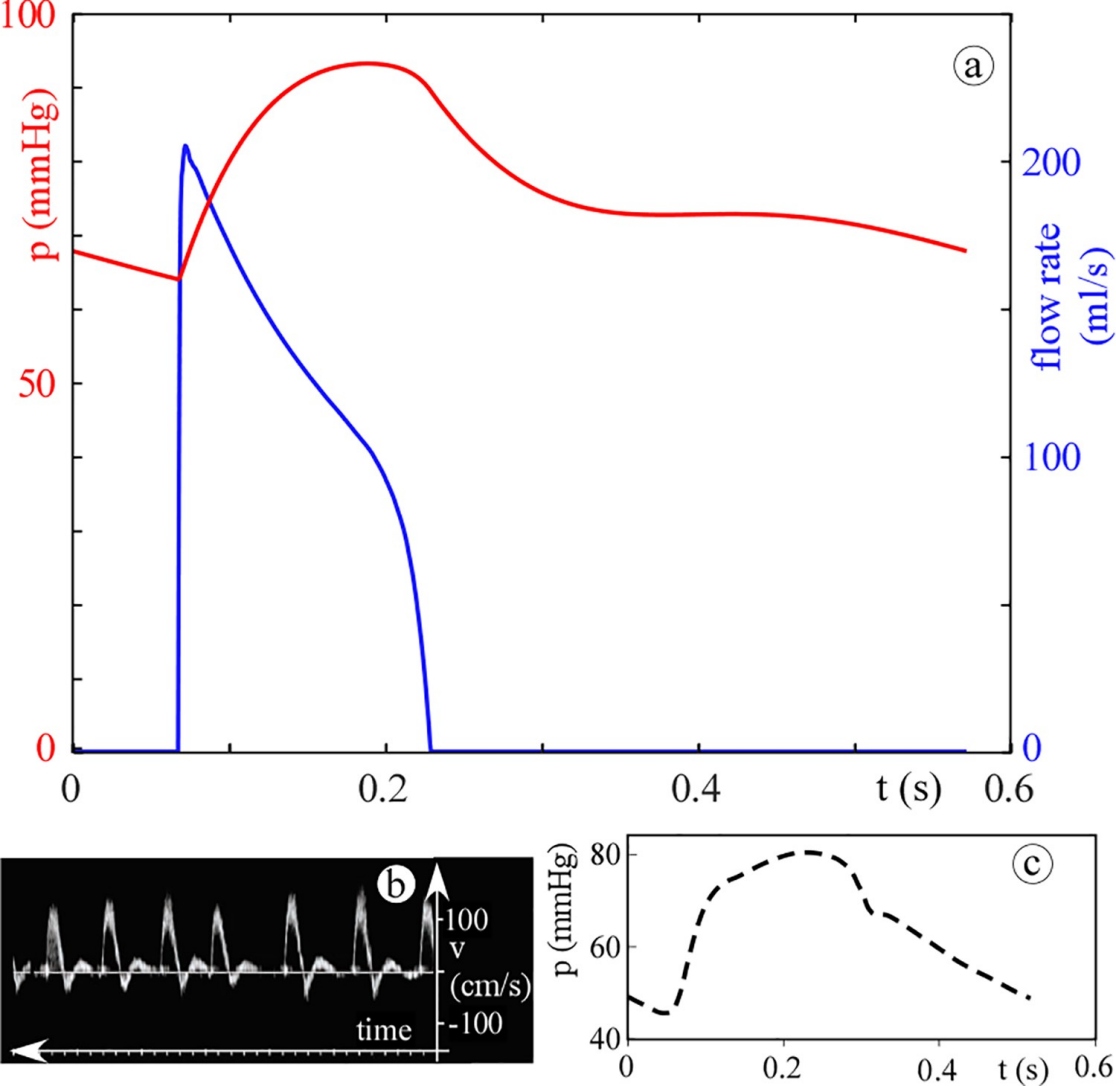
Panel (a): 0D model results for the physiologic case (aortic pressure, red curve; aortic flow rate, blue curve). Panels (b) and (c): published *in-vivo* data (aortic Doppler velocity waveform [19] and aortic pressure [20]). Note that, by assuming a constant valve area, the velocity waveform resembles the flow one except for the absolute values and the scale.

**Fig 4:**
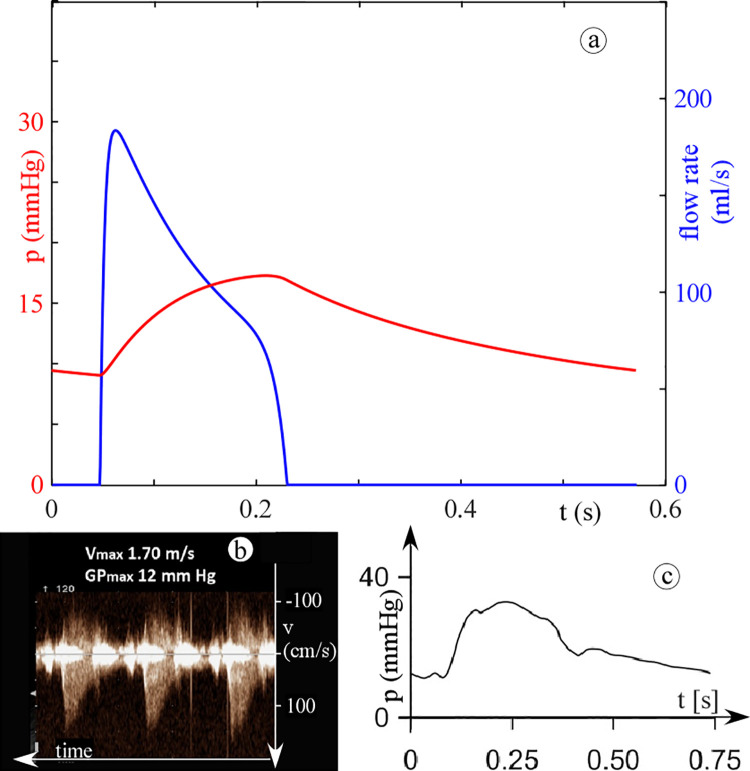
Panel (a): 0D model results for the physiologic case (pulmonary arterial pressure, red curve; pulmonary flow rate, blue curve). Panels (b): [22] and (c): [23] published data (pulmonary Doppler velocity waveform and pulmonary arterial pressure, respectively). Note that, by assuming a constant valve area, the velocity waveform resembles the flow one except for the absolute values and the scale.

Figs 3 and 4 show the agreement between the computed pressure and flow rate and those given in literature for both the systemic and pulmonary circulation. In particular, Fig 3 shows the physiological aortic pressure and flow rate calculated by the 0D model together with published *in vivo* measurements obtained in paediatric subjects [19, 20]. There is a good agreement in terms of waveform shapes, and the computed and *in vivo* mean aortic pressure are about 70 and 60 mmHg, respectively. Additionally, from the peak flow rate and velocity of 200 mL/s (Fig 3A) and 150 cm/s (Fig 3B), respectively, an aortic diameter of about 1.3 cm is found. This value agrees with the *in vivo* measurement of Hegde *et al*. [21] for a child with a BSA of 0.55 m^2^. Similarly, Fig 4 shows the physiological waveforms for the pulmonary circulation compared to reference data [22, 23]. Note that, for the pulmonary circulation, the references are not age specific. However, the computed waveforms well resemble the physiological characteristics. Notice also that the valve backflow is not computed by the model since only the forward flow is considered (on/off diodes). This simplification does not affect the results since we focused on the ventricular obstructions severity i.e., in the ejection phase only.

**Fig 5:**
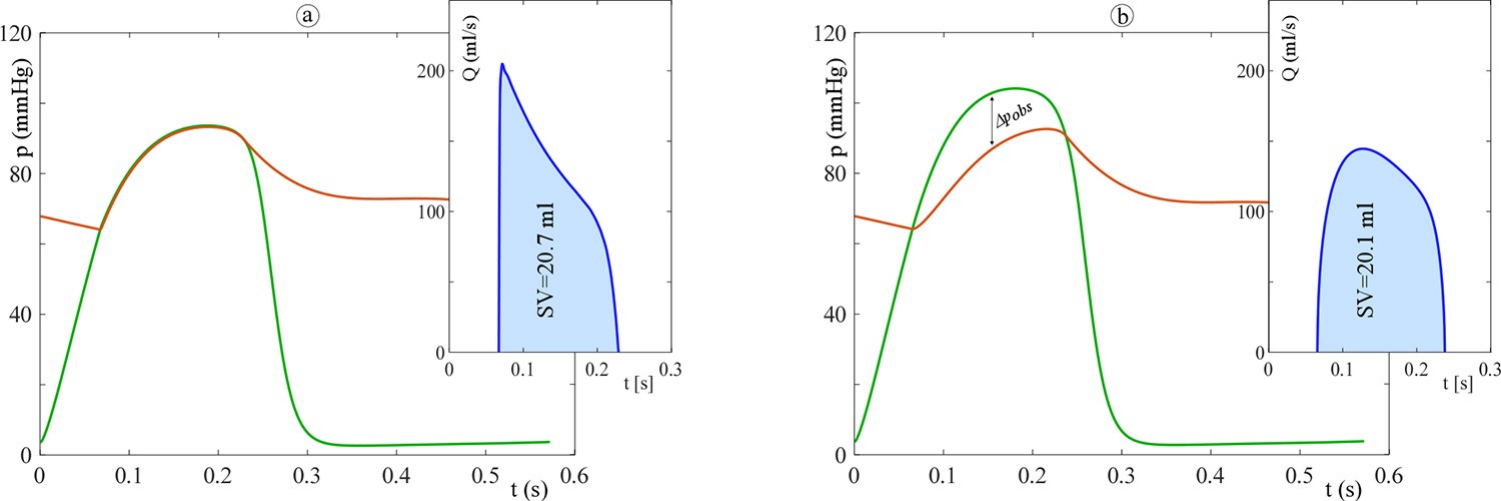
Left ventricle pressure (green), aortic pressure (red), and aortic flow rate (blue) as calculated by the model for the physiologic case (panel (a)) and the case of obstruction located in the LVOT with *A*_*free*_/*A* = 0.525 (panel (b)).

Fig 5 compares the results obtained from the model for the physiologic case (panel (a)) and, as an example of obstruction in the LVOT, for the *A*_*free*_/*A* = 0.525 case (panel (b)). As it is evident in Fig 5B, when the obstruction is included in the model at any level in the LV, the LV pressure increases, maintaining the aortic pressure unchanged and resulting in a pressure gradient (Fig 5A). Similar findings are reported by De Vecchi *et al*. [24] for the case of LVOTO after transcatheter mitral valve replacement in adults.

**Fig 6:**
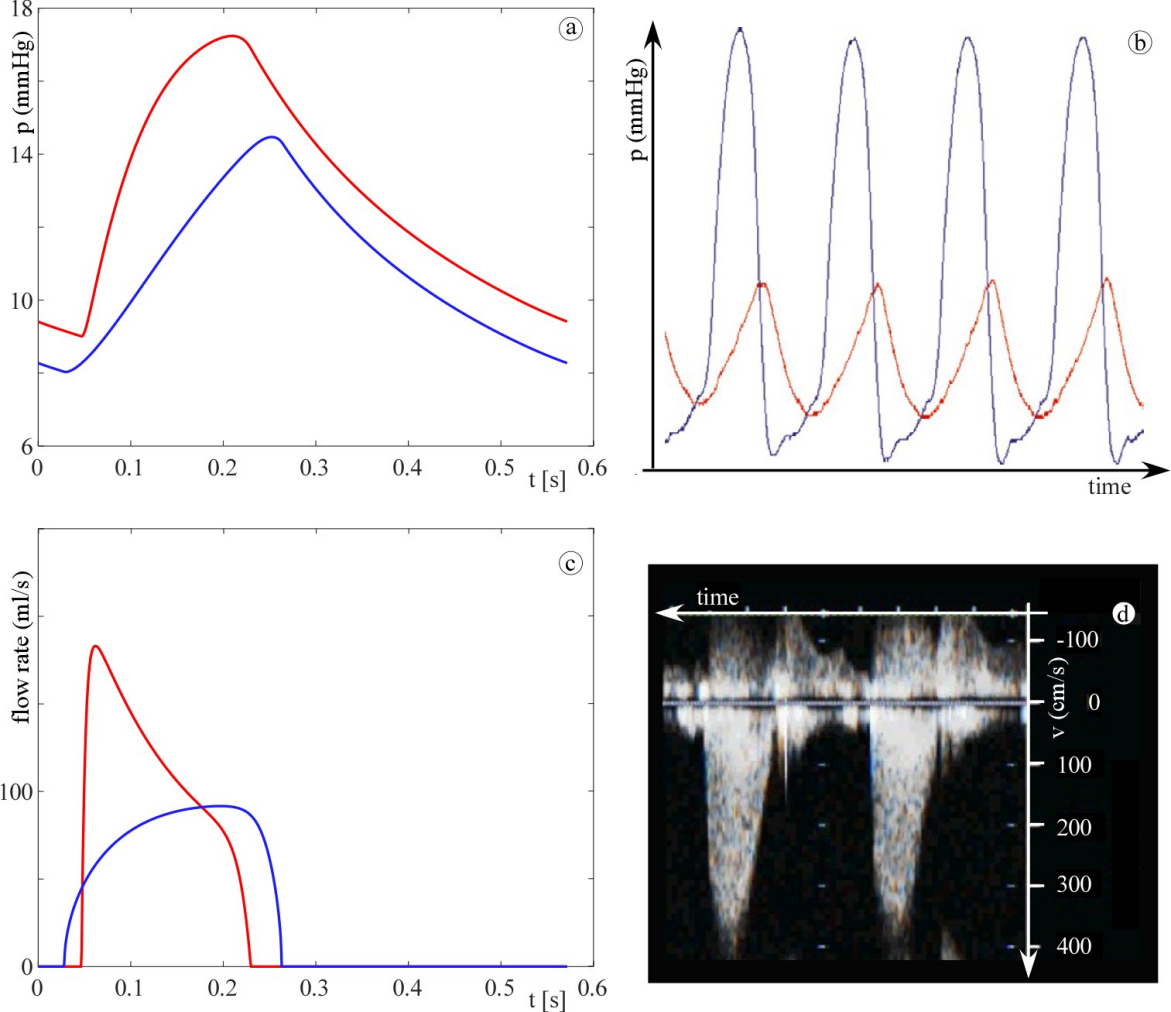
Pulmonary arterial pressure and flow rate. (a) computed pulmonary arterial pressure, (b) stenotic reference data (blue and red, right ventricular and pulmonary arterial pressure, respectively) [25], (c) computed pulmonary flow rate, and (d) stenotic reference data [22]. For the computed waveforms: in red, the healthy condition, and in blue, the stenotic case.

Differently, RVOT obstruction does alter the shape of the pulmonary arterial pressure with the stenotic case exhibiting the typical delayed, lowered, and triangular appearance as stated by Butera *et al*. [25] (see Fig 6). For what the valve flow is concerned, the presence of the obstruction (either in LVOT or in RVOT) also significantly affects the shape of the ejected flow rate waveform (Figs 5 and 6). The steepness of the acceleration branch smooths, the branch peaks at a lower flow rate, the time of peak is delayed in the ejection period, which has a longer duration, and, as a result, the waveform moves from a rather triangular to a bell shape. It is worth noting that such a behaviour is recognized to occur in presence of aortic and pulmonary valve stenosis [26–30], which further confirms the robustness of the model in computing obstructed flows characteristics.

**Fig 7:**
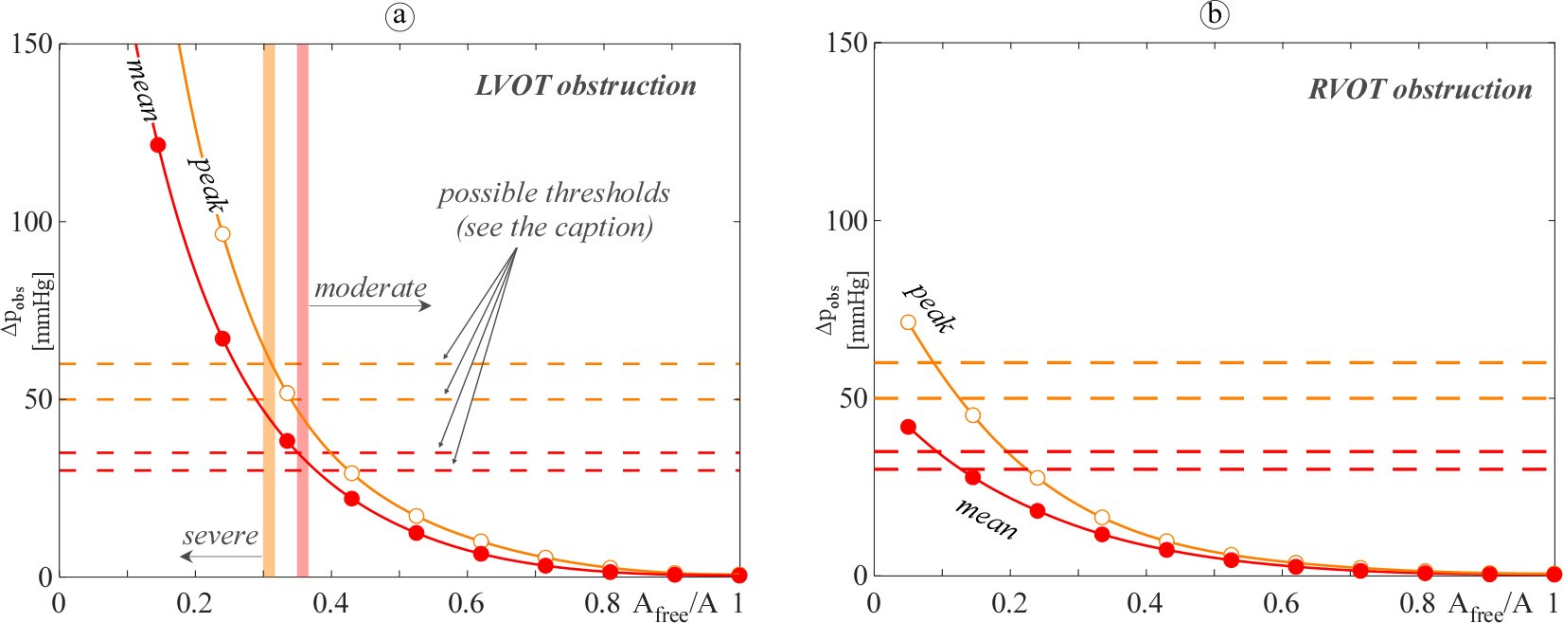
Computed mean (red filled dotted line) and peak (orange open dotted line) pressure gradient for (a) LVOT and (b) RVOT. Dashed lines depict possible thresholds for moderate/severe obstruction, inferred from the literature for the case of a tumoral obstructive condition as it follows: mean gradient 30 mmHg [32] and 35 mmHg [31], red lines; peak gradient 50 mmHg [33] and 60 mmHg [31], orange lines.

Fig 7 shows the behaviour of the mean and the peak pressure gradient across the obstruction as a function of the narrowing ratio *A*_*free*_/*A* (left panel: LVOT obstructions; right panel: RVOT obstructions). Possible threshold values for the mean (peak) pressure gradient as inferred from the literature for the case of a tumoral obstructive condition are also drawn for comparison [31–33]. As expected, both the mean and the peak pressure gradients, Δ*p*_*obs*,*mean*_ and Δ*p*_*obs*,*peak*_, increase as the obstruction becomes more severe. Obstructions located in the LVOT or in the RVOT result in similar exponential behaviour, but a larger rate of change of the gradient with *A*_*free*_/*A* diminishing is found for left obstructions i.e., LVOT obstructions seem to impact the heart pressure state more heavily than RVOT ones. This behaviour might be related to the recognized capability of the RV to adapt to pulmonary valve stenosis i.e., pressure overload conditions [34].

Results shown in Fig 7 can be of help in estimating the degree of obstruction severity by comparing model computed gradients to threshold values measured in clinical practice. When the R/LVOT obstruction is caused by a mass (i.e., cardiac tumour), reference values for the limit between moderate and severe obstruction can be inferred from the recent literature. Dyspnoeic children with left mean pressure gradient greater than 30 mmHg are reported by Walter *et al*. [32]; the case of a patient with left obstruction reported as moderate, mean gradient 35 mmHg and peak gradient 60 mmHg is described in [31]; finally, Padalino *et al*. [33] define as significant a left or right obstruction with peak gradient larger than 50 mmHg. The comparison of the simulation’s outputs with the above clinical thresholds (Fig 7) highlights that moderate/severe obstruction is found for *A*_*free*_/*A* ≈ 0.3–0.38 and *A*_*free*_/*A* ≈ 0.1–0.15 for LVOT and RVOT obstruction, respectively. Our results show that the computed right critical narrowing ratio might seem so small to be unrealistic. However, this is in agreement with what reported in the clinical field by Foschi *et al*. [34] who stated that “*In moderate-to-severe pulmonary valve stenosis*, *patients remain asymptomatic until adulthood*”. Furthermore, the relationships obtained between the gradient and the level of the obstruction may also provide the surgeon with advice in regards to the degree of myocardial resection that she/he should perform to significantly reduce the pressure gradient. For instance, in the LVOT obstruction case, a resection leading to *A*_*free*_/*A* of about 0.45 is required to diminish the mean pressure gradient below 20 mmHg, which is typically considered the gold standard. Hence, the proposed model is a first step towards a tool that can potentially help in the clinical management of patients. Indeed, there are only few engineering works focused on models for obstructive diseases, especially for studying VOT obstruction of the right ventricle in paediatric patients, which has a high incidence, but is less studied. Thus, models that help in extending knowledge of these pathologies can help in improving both diagnosis and treatment. Indications of the percentage of obstructions can help in identifying the severity of the pathology, and in case of obstructive masses, a suggestion of the amount of mass to be resected can be derived.

**Fig 8:**
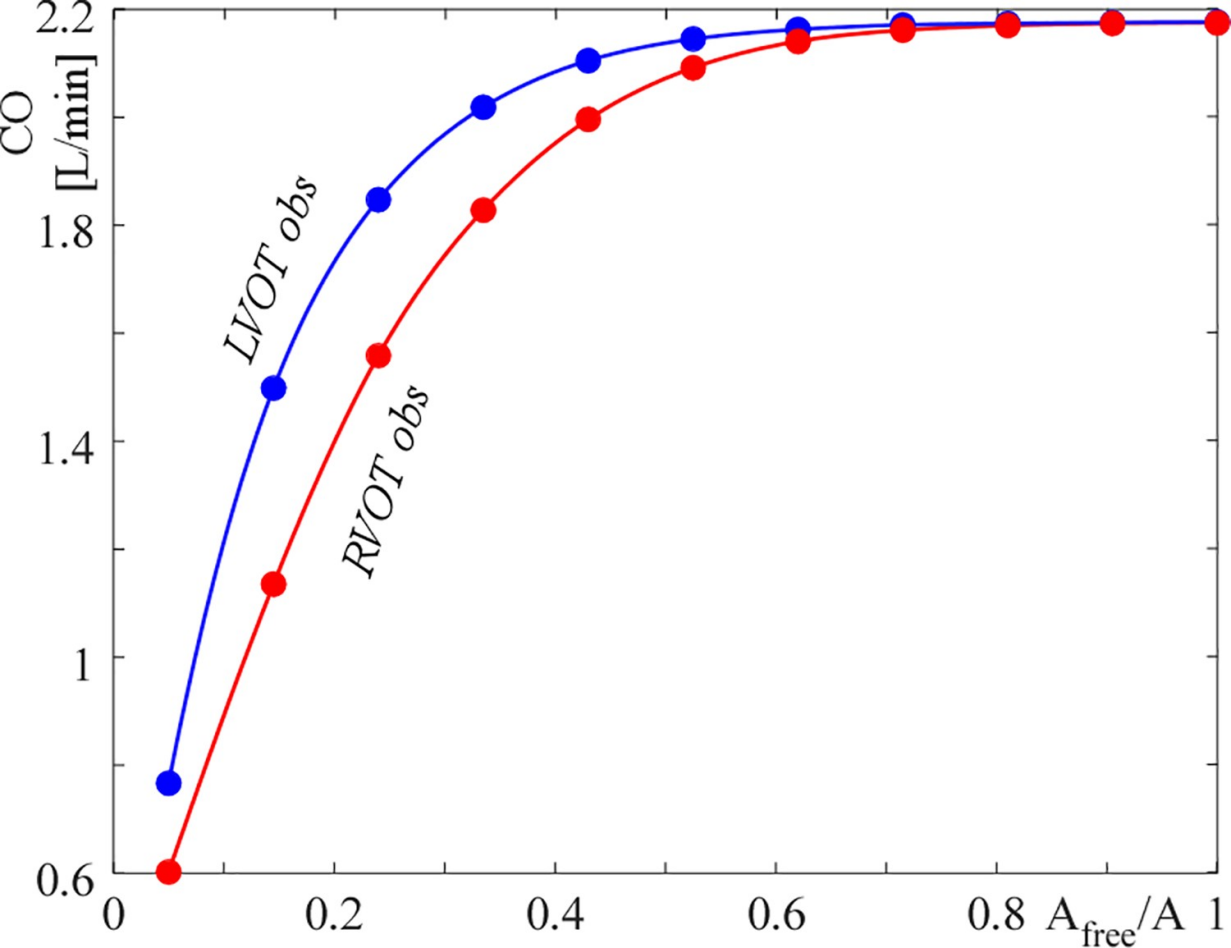
Cardiac output (CO) reduction as the obstruction worsens for LVOT (blue) and RVOT (red) obstructions.

When focusing the attention on the obstruction’s effects on the cardiac output (Fig 8), it turns out that, RVOT obstructions affect the CO more than what LVOT ones do. In particular, CO = 2.0 L/min (i.e., almost normal) and CO = 0.9 L/min (i.e., significantly depressed) is found when the critical pressure gradient (mean gradient about 30–35 mmHg) is attained in the left and in the right outflow tract, respectively. The model hence captures the recognized lower efficiency of the right ventricle to suitably respond to increased afterload [35, 36], suggesting that critical conditions should be defined in terms of cardiac output rather than of pressure gradient when it is the right outflow tract to be obstructed.

**Fig 9:**
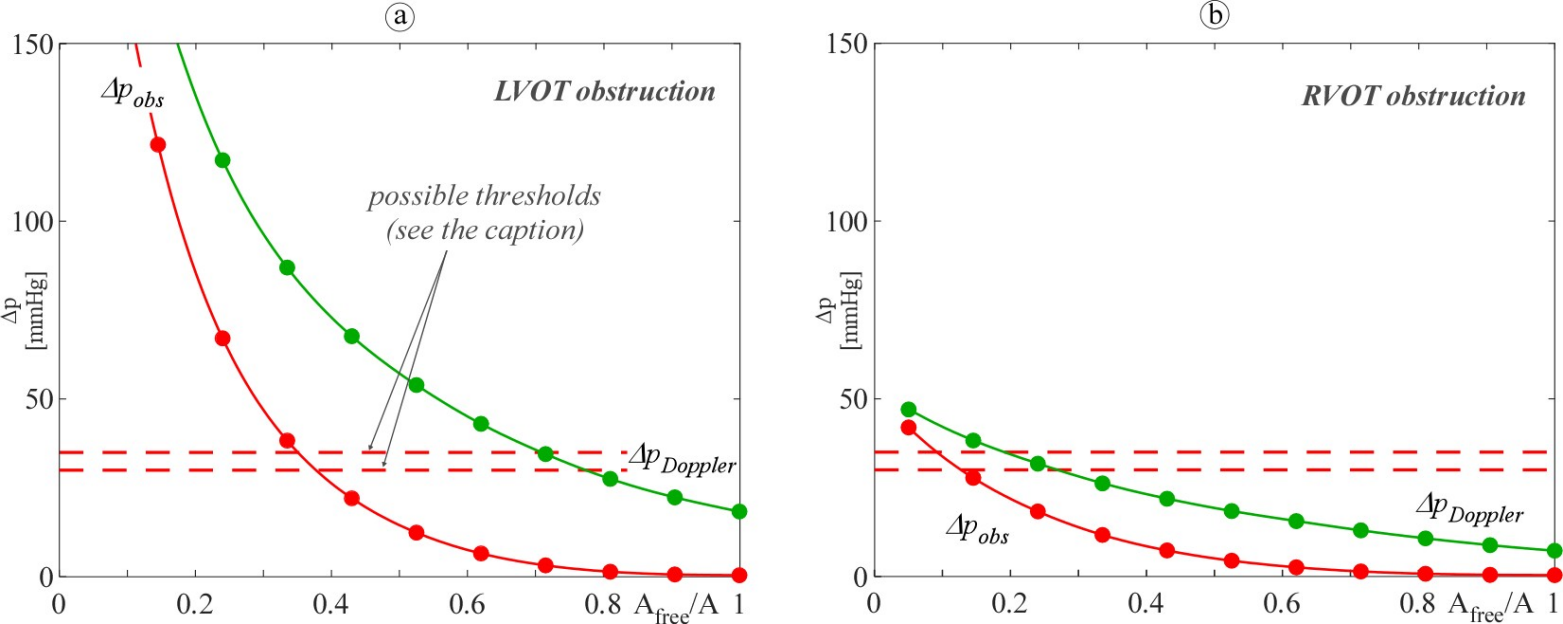
Computed mean pressure gradient with (red filled dotted line, Δ*p*_*obs*_) and without (green filled dotted line, Δ*p*_*Doppler*_) the pressure recovery effect for (a) LVOT and (b) RVOT. Red dashed lines depict possible mean gradient thresholds for moderate/severe obstruction, inferred from the literature for the case of a tumoral obstructive condition as it follows: 30 mmHg [32] and 35 mmHg [31].

Finally, we further harnessed the model to point out the pressure recovery effect on the evaluation of VOT obstruction, i.e., we compared Δ*p*_*obs*_ and Δ*p*_*Doppler*_ (Fig 9). We recall that the two above gradients do/do not take into account the pressure recovery, respectively, and that Δ*p*_*Doppler*_ is the gradient clinically evaluated by Doppler echocardiography. As expected in a paediatric patient, the mean Δ*p*_*Doppler*_ (i.e., the ‘pre-recovery’ gradient) is larger than the mean Δ*p*_*obs*_ (i.e., the ‘post-recovery’ gradient) for both LVOT and RVOT obstruction, and the largest difference falls around moderate obstructions [37] i.e., where the accuracy of the clinical evaluation of the lesion severity is pivotal for the patient management. However, the discrepancy between the two gradients is found to be much more pronounced for LVOT rather than RVOT obstruction. In particular, for the LVOTO case the Doppler gradient overpasses the possible moderate/severe thresholds for values of *A*_*free*_/*A* even double than those found for Δ*p*_*obs*_ (*A*_*free*_/*A* around 0.7 vs *A*_*free*_/*A* around 0.35, respectively). On the contrary, for the RVOTO case both the computed gradients remain under the thresholds up to *A*_*free*_/*A* 0.2–0.3. Such a result suggests that not only the pressure recovery affects right lesions much less than the left ones but also that misclassification of lesion severity by Doppler is more likely to occur when the occlusion is located in the left rather than in the right side of the heart. Thus, tools as the one here proposed, which is capable of taking into account the pressure recovery effect, might be of particular help in sparing cardiac catheterization, i.e., invasive procedures, to left side diseased patients.

### Limitations and future developments

The adopted shape of the obstruction, described as an ideal round, annular, and concentric narrowing, is the main limitation of our study. Actually, real obstructions might present an irregular geometrical structure, hence our assumption might underestimate the actual value of the loss coefficient *K*_*obs*_ in Eq (2). However, a sensitivity analysis of model results to the factor *f*_*shape*_ was performed to highlight the relevance of obstacle’s shape effects. Calculations show that accounting for a larger contraction of the proximal flow due to the real shape of the obstruction i.e., a larger *k*_*obs*_ for the given *A*_*free*_/*A*, leads to moderate variations of model’s outputs for the left case: the critical obstruction (mean and peak Δ*p*_*obs*_ equal to about 35 and 50 mmHg, respectively) shifts to *A*_*free*_/*A* = 0.48, with cardiac output still around 2.0 L/min. For the right case, the effects of *f*_*shape*_ are found to be so small to be undiscernible.

It is also worth noticing that, differently from our assumption, both the right and left VOT shape has been recognized to be more elliptical than circular [38–41]. Our assumption might hence lead to some mis-estimation of stenotic areas. Moreover, VOT geometry might not be constant during the ejection phase; however, to the best of our knowledge, such a behaviour is not sufficiently described by clinical data to be adequately modelled yet.

The absence of adaptive and remodelling processes which take place in physiological conditions is another limitation of our work. For example, an increase in systemic vascular resistance may counterbalance the effects of the reduced stroke volume and aortic pulse pressure in the case of LVOT obstructions. These aspects will be included in more detail in future work.

Moreover, to further corroborate the model here presented, acquisition and analysis of *in vivo* morpho-hemodynamic data of the obstructed outflow tract, together with obstruction classification based on clinical examination of patients, is strongly recommended. Future work will hence also focus on the creation of a database listing imaging, echo, catheter and other data, and on the comparison of *in vivo* data to model’s outputs to optimize the estimation of *f*_*shape*_ and *K*_*obs*_ for a given size, shape, and type of R/L VOTO.

Finally, it is worth noting that the model has been here applied to a reference paediatric case, but it can be adopted also to reproduce different populations/subgroups/specific patients by properly calibrating hemodynamic parameters. Similarly, by considering sex-specific parameters, sex-related characteristics can be accounted for [17]. For what subgroups examination is concerned, the model can help in investigating the effects of the wide inter-subject variability of the anatomy and pathophysiology. In order to give a preliminary insight in that direction, we run the model for a 10 years-old reference child. Results have shown that both the pressures and the CO are less affected in older than in younger patients by a given *A*_*free*_/*A*, thus suggesting that older patients better sustain VOT obstructions, in agreement with Singh [42]. It can be speculated that such a result depends on the lower vascular resistances of older patients [18]. Patient-specific simulations are also one of our future goals. For this purpose, non-invasive techniques will be used (e.g., imaging, echo, MR) to evaluate the patient-specific morphology and hemodynamics from which patient-specific parameters will be derived.

## Conclusions

Obstruction of the R/L ventricular outflow tract is a common disease, in particular in the paediatric population. Clinical decision-making in the management of patients remains controversial in a significant number of cases. Nonetheless, engineering researches devoted to model ventricular outflow tract obstructive disease are still limited. For this reason, we developed a simple lumped parameter model of hemodynamics in presence of a R/LVOT obstruction, able to give indications on the most significant waveforms, pressure gradient, and cardiac output alterations produced by a given obstruction size.

The lack of clinical data did not allow detailed comparative analysis with physiological measurements of obstructed flows, but model results proved in very good agreement with trends observed in clinical setting thus corroborating the idea that the proposed approach is useful to understand the clinical deterioration of patients and can contribute, with further validation, to the creation of a tool applicable in the clinical practice.

Indeed, the simulations highlighted that the left and right circulations give significantly different response to VOT obstructions. In particular, model predictions suggested that, for the case of right VOTO, the clinical assessment should mainly focus on the CO reduction rather than on the ventriculo-arterial pressure gradient increase. Moreover, the capability of the model of taking into account the pressure recovery effect showed that the model has the potential to become a tool alternative to catheterization, thus preserving borderline patients from invasive procedures. Finally, the design of surgical treatments for obstruction mitigation can benefit from model indications of VOT free area to be restored to accomplish an acceptable pressure gradient/cardiac output.

## Supporting information

S1 File Model of obstructed flow. (DOCX)

S2 File Sensitivity analysis of model results to the shape factor *f*_*shape*_. (DOCX)

S3 File Model of the complete circulation. (DOCX)

S4 File Parameters’ values. (DOCX)

S5 File Global sensitivity analysis. (DOCX)
