# Supplementary material for: Ventricular outflow tract obstruction: An in-silico model to relate the obstruction to hemodynamic quantities in cardiac paediatric patients
Source: PLoS One. 2021 Oct 15;16(10):e0258225. doi: 10.1371/journal.pone.0258225 (PMC8519477; doi:10.1371/journal.pone.0258225)
Supplement: S1 File — (DOCX) [file pone.0258225.s001.docx]

# **S1** Model of obstructed flow

The fluid dynamics scheme reported in Susin [7] to describe the local flow across a stenotic valve was here adopted to model the hemodynamics in the obstructed R/LVOT, but with some adaptations. In particular, in the present case the geometry of the blood jet across the obstruction is considered invariant in time and the quasi-steady state is assumed for the instantaneous flow rate $Q(t)$. As a consequence, the inertia contribution to the transtenotic pressure drop vanishes, i.e. the drop reduces to the head loss term only, which is the footprint of the viscous dissipation due to distal vortices and represents the primary contribution to the stenotic pressure gradient, and in particular to its mean value in the ejection period [7]. Moreover, an ideal round annular obstruction rather than an orifice plate is here considered for the obstacle, which allows to assume that the upstream streamlines smoothly adapt to the narrowing and the vena contracta (i.e., the smallest jet area) establishes at the $A_{free}$ section (see Fig. 2). Hence, the effective orifice area of the stenotic valve flow in Susin [7] here coincides with $A_{free}$ and the model of the pressure drop finally reads

$\Delta p_{obs}=\frac{\rho Q^{2}}{2}\left( \frac{1}{A_{free}}-\frac{1}{A} \right)^{2}$ (S1.1)

where A is the area of the circular pipe representing the R/LVOT, and $\left( \frac{1}{A_{free}}-\frac{1}{A} \right)^{2}$ is the so-called loss coefficient.

However, real obstructions can be even quite far from the ideal round morphology, so that the vena contracta establishes downstream than the $A_{free}$ section and the minimum jet area is smaller than $A_{free}$ (Fig. 1). To account for such a circumstance, the loss coefficient must be increased by a shape factor $f_{shape}$ larger than 1, and the pressure drop $\Delta p_{obs}$ rewritten as given in Eq. (2) (or in compact form as in Eq. (1)).
