# Supplementary material for: Ventricular outflow tract obstruction: An in-silico model to relate the obstruction to hemodynamic quantities in cardiac paediatric patients
Source: PLoS One. 2021 Oct 15;16(10):e0258225. doi: 10.1371/journal.pone.0258225 (PMC8519477; doi:10.1371/journal.pone.0258225)
Supplement: S2 File — (DOCX) [file pone.0258225.s002.docx]

**S2 Sensitivity analysis of model results to the shape factor** $\boldsymbol{f}_{\boldsymbol{shape}}$

The simulations were conducted assuming $f_{shape}$= 1 i.e., considering that the blood flow approaching the obstruction contracts up to the minimum area $A_{free}$. However, the real contraction may be larger as an effect of the actual morphology of the obstruction, as the literature on 'orifice flows' has widely demonstrated (see, for example, [16]). In that case, the minimum cross-sectional area of the flow through the obstruction is smaller than $A_{free}$, and can be expressed as ${c_{c}A}_{free}$, where $c_{c}$ is the contraction coefficient [16]. As a result, the $f_{shape}$ factor assumes values larger than 1. To estimate a realistic range of variability of $f_{shape}$ it is convenient to rewrite the coefficient $K_{obs}$ given in Eq. (2) as

$K_{obs}=\frac{\rho}{2}\left( \frac{1}{{c_{c}A}_{free}}-\frac{1}{A} \right)^{2}$ (S2.1)

which immediately provides the relationship between the shape factor and the size of the contraction

$f_{shape}=\left( \frac{A-c_{c}A_{free}}{c_{c}A-c_{c}A_{free}} \right)^{2}$. (S2.2)

The behaviour of the contraction coefficient as a function of the ratio between the orifice area and the area of the pipe i.e., $A_{free}/A$ has been widely investigated in the literature for a number of different orifice morphology. Among the others, the relationship proposed in Ferrari [43], which reads

$c_{c}=\left[ 1+\frac{2}{\pi}\left( \frac{1}{c_{c}{A_{free}}^{0.5}}-c_{c}{A_{free}}^{0.5} \right)atan\left( c_{c}{A_{free}}^{0.5} \right) \right]^{-1}$. (S2.3)

proves to be in excellent agreement with the results reported in [44] for the case of flow through stenotic valve orifices and was here adopted.

Fig. S2.1 shows the behaviour of $c_{c}$ and $f_{shape}$ as obtained according to Eq. (S2.3) and Eq. (S2.2), respectively, and assumed as range for the sensitivity analysis.


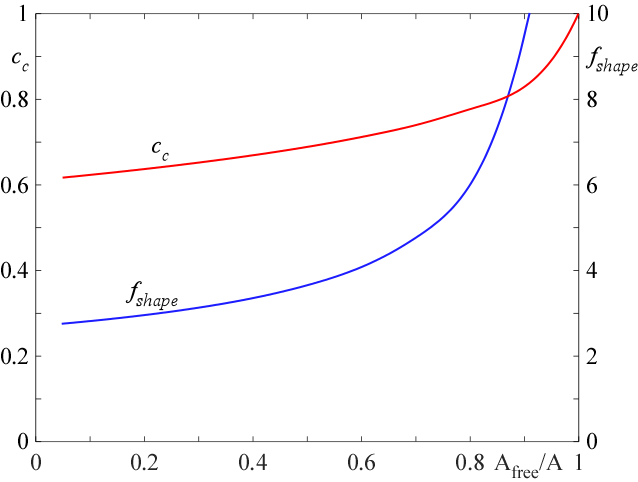


**Fig. S2.1.** Contraction coefficient $c_{c}$ and shape factor $f_{shape}$ as a function of $A_{free}/A$, adopted for the sensitivity analysis.

**References**

[43] Ferrari A. Fluid dynamics of acoustic and hydrodynamic cavitation in hydraulic power systems. Proc R Soc A Math Phys Eng Sci. 2017;473. doi:10.1098/rspa.2016.0345

[44] Garcia D, Pibarot P, Landry C, Allard A, Chayer B, Dumesnil JG, et al. Estimation of aortic valve effective orifice area by Doppler echocardiography: Effects of valve inflow shape and flow rate. J Am Soc Echocardiogr. 2004;17: 756–765. doi:10.1016/j.echo.2004.03.030
