# Supplementary material for: Ventricular outflow tract obstruction: An in-silico model to relate the obstruction to hemodynamic quantities in cardiac paediatric patients
Source: PLoS One. 2021 Oct 15;16(10):e0258225. doi: 10.1371/journal.pone.0258225 (PMC8519477; doi:10.1371/journal.pone.0258225)
Supplement: S3 File — (DOCX) [file pone.0258225.s003.docx]

# **S3 Model of the complete circulation**

The lumped parameter methodology allows to analyse the global hemodynamics using a limited number of parameters, the circulation model is shown in Fig. S3.1. The model is composed by pulmonary and systemic circulations, the latter divided into the main organs: liver, gastro-intestinal tract, kidneys, upper and lower bodies. Each vascular portion was represented by an appropriate combination of Windkessel parameters: capacitor (C), resistance (R), and inductance (L), that reproduce the wall deformability, the viscous resistance and the blood inertia, respectively. Particularly, the main arteries and veins were represented as reported in Fig. S3.2a, considering R, C, and L, whereas the main organs were subdivided in arterial-arteriolar, vascular bed, and venous sections. The arteries and venous side were reproduced considering R and C, and the vascular bed of each simulated organs consisted in a resistance (Fig. S3.2b). The governing equations for blood flow are obtained imposing the mass and momentum conservation laws. These, in the 0D scheme, reduce to

$\left\{ \begin{matrix} C\frac{{dp}_{j}}{dt}+Q_{j+1}=Q_{j} \\ L\frac{{dQ}_{j+1}}{dt}+RQ_{j+1}-p_{j}={-p}_{j+1} \end{matrix} \right.$ (S3.1)

with ${(p_{j},Q}_{j})$ and $(p_{j+1},Q_{j+1})$ being the inlet/outlet pressure and flow rate, respectively.


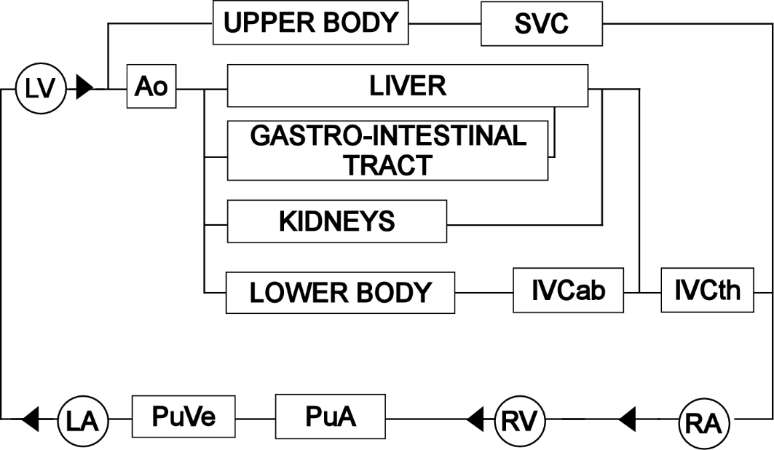


**Fig. S3.1.** Physiological circulation model. The model is composed by the aorta, Ao, the liver, the gastro-intestinal tract, the kidneys, the upper and lower body, the abdominal and thoracic inferior vena cava, IVCab and IVCth, respectively, the superior vena cava, SVC, the pulmonary arteries and veins, PuA and PuVe, respectively, and, the four heart chambers: left ventricle, LV, left atrium, LA, right ventricle, RV, right atrium LA, and ►, heart valves.


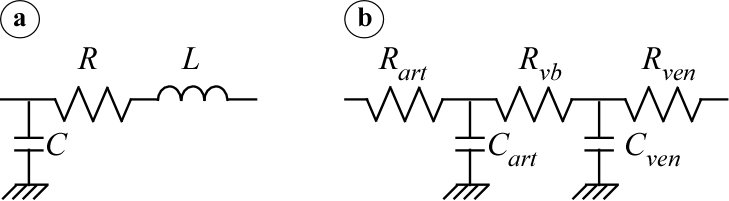


**Fig. S3.2.** Lumped scheme for (a) great vessel, and (b) organs of interest. R, resistance, C, capacitance, L, inductance, R_art_, arterial-arteriolar resistance, R_vb_, vascular bed resistance, R_ven_, venous resistance, C_art_, arterial-arteriolar compliance, and C_ven_, venous compliance.

The four heart chambers were modelled according to its anatomical structure: the contracting/relaxing action was simulated by a time-varying elastance function derived from the recognized myocardial elastance theory [45] as in [46]. Thus, the pressure within each heart chamber ($p\left( t \right)$) reads

$p\left( t \right)=E(t)(V\left( t \right)-V_{p=0})$ (S3.2)

where $E(t)$ is the time-varying elastance, $V\left( t \right)$ is the chamber volume and $V_{p=0}$ is the unstressed volume at zero pressure. $E(t)$ was simulated exploiting the ‘two-Hill’ function which considers pressure variations due to myofibers activation and volume variations within the chamber as

$E\left( t \right)=k\left( \frac{g_{1}}{1+g_{1}} \right)\left( \frac{1}{1+g_{2}} \right)+E_{min}$. (S3.3)

$k$ is a scaling factor used to guarantees that $\max\left( E\left( t \right) \right)=E_{max}$ i.e., the maximal chamber elastance, as $k=\frac{E_{max}-E_{\min}}{\max\left[ \left( \frac{g_{1}}{1+g_{1}} \right)\left( \frac{1}{1+g_{2}} \right) \right]}$, $g_{1}=\left( \frac{t-t_{onset}}{\tau_{1}} \right)^{m_{1}}$ and $g_{2}=\left( \frac{t-t_{onset}}{\tau_{2}} \right)^{m_{2}}$. The chamber’s contraction and relaxation are determined by the first and the second Hill functions, respectively; whereas, $t_{onset}$ is the time-shift for the atrial contraction, $\tau$ controls the relative appearance in time within the heartbeat, and $m$ governs the steepness.

The four heart valves i.e., aortic ($\mathrm{Ao}$) and mitral ($\mathrm{Mv}$) valves in the left heart and pulmonary ($\mathrm{Pv}$) and tricuspid ($\mathrm{Tv}$) valves in the right heart, were simulated considering their resistance effect. Therefore, the flow throughout the valve ($Q$) is

$Q(t)=\Delta p(t)/R_{v}$ (S3.4)

with ∆p the pressure difference across the valve and $R_{v}$ the valve resistance. The valves open and close instantaneously according to the pressure gradient across each of them i.e., the valve dynamics was neglected.

**References**

[45] Suga H, Sagawa K, Shoukas AA. Load independence of the instantaneous pressure-volume ratio of the canine left ventricle and effects of epinephrine and heart rate on the ratio. Circ Res. 1973;32: 314–322. doi:10.1161/01.RES.32.3.314

[46] Mynard JP, Smolich JJ. One-Dimensional Haemodynamic Modeling and Wave Dynamics in the Entire Adult Circulation. Ann Biomed Eng. 2015;43: 1443–1460. doi:10.1007/s10439-015-1313-8
