# Supplementary material for: Ventricular outflow tract obstruction: An in-silico model to relate the obstruction to hemodynamic quantities in cardiac paediatric patients
Source: PLoS One. 2021 Oct 15;16(10):e0258225. doi: 10.1371/journal.pone.0258225 (PMC8519477; doi:10.1371/journal.pone.0258225)
Supplement: S4 File — (DOCX) [file pone.0258225.s004.docx]

**S4 Parameters’ Values**

To reproduce the physiological hemodynamics, the 0D model was adequately calibrated as previously reported [17]. The total peripheral resistance ($R_{tot}$) for any systemic compartment represented by Fig. S3.2b was calculated by considering a perfusion pressure (i.e., mean pressure that perfuses the systemic organs) of 65 mmHg [47], a cardiac output (CO) of 2.1 L/min [48], and the percentage of CO that supplies each organ [48–50]. Each resistance is computed considering $R_{art}=5\%R_{tot}$, $R_{vb}=92\%R_{tot}$ , and $R_{ven}=3\%R_{tot}$ i.e., simulating the greater dissipation effect in the microvascular resistance. Note, a fine tuning of the portal system resistance was performed to represent the flow from the portal vein to the liver [17]. Given the resistance values, the corresponding arterial compliance ($C_{art}$) was computed harnessing the time constant of the systemic arterial system, $\tau$= 0.81 s, as $C_{art}=\tau/R_{vb}$ [49]; whereas the venous compliance ($C_{ven}$) was considered 30 times greater than $C_{art}$ [48]. For the great vessels, resistance values in agreement with the allometric law of Baretta *et al.* [51] were imposed, adjusting the compliances to obtain the total compliance in the range of 2.0-2.5 mL/mmHg/kg [49]. For the pulmonary circulation, the compliance values were adjusted from [52], and the resistance values were derived as described above considering a pulmonary time constant of $\tau$ = 0.2 s [49]. Inductance values were taken from [53] and adjusted considering the allometric law of Baretta *et al.* [51]. For the heart parameters, values from [46,49] were imposed (reported in Table S4.1). Particularly, $V_{p=0}$ of Eq. (S3.2) was adjusted to obtain the specific paediatric hemodynamics, whose values were then kept constant during the simulation of the left/right ventricular outflow tract obstruction. Note that, the methodology here described to calibrate the paediatric model derives from the calibration of adult models. However, since the input values result from paediatric-specific parameters (e.g., CO of 2.1 L/min) and adjustments by the allometric laws were performed, the methodology was reliable also for the paediatric population.

**Table S4.1.** Heart parameters values.

|  | **LV** | **RV** | **LA** | **RA** |
| --- | --- | --- | --- | --- |
| $\boldsymbol{E}_{\boldsymbol{max}} \left[ \boldsymbol{mmHg}/\boldsymbol{mL} \right]$ | 2.8 | 0.45 | 0.13 | 0.09 |
| $\boldsymbol{E}_{\boldsymbol{min}} \left[ \boldsymbol{mmHg}/\boldsymbol{mL} \right]$ | 0.07 | 0.035 | 0.09 | 0.045 |
| $\boldsymbol{V}_{\boldsymbol{p=0}} \left[ \boldsymbol{mL} \right]$ | -20 | -20 | -30 | -40 |
| $\boldsymbol{\tau}_{\boldsymbol{1}} \left[ \boldsymbol{s} \right]$ | 0.269*T* | 0.269*T* | 0.110*T* | 0.110*T* |
| $\boldsymbol{\tau}_{\boldsymbol{2}} \left[ \boldsymbol{s} \right]$ | 0.452*T* | 0.452*T* | 0.180*T* | 0.180*T* |
| $\boldsymbol{m}_{\boldsymbol{1}} \left[ \boldsymbol{-} \right]$ | 1.32 | 1.32 | 1.99 | 1.99 |
| $\boldsymbol{m}_{\boldsymbol{2}} \left[ \boldsymbol{-} \right]$ | 21.9 | 21.9 | 11.2 | 11.2 |
| $\boldsymbol{t}_{\boldsymbol{onset}} \left[ \boldsymbol{s} \right]$ | 0 | 0 | 0.85*T* | 0.85*T* |
| $\boldsymbol{R}_{\boldsymbol{v}} \left[ \boldsymbol{mmHg\cdot s}/\boldsymbol{mL} \right]$ | 0.0036 | 0.0036 | 0.036 | 0.036 |

**References**

[47] Cattermole GN, Leung PYM, Mak PSK, Chan SSW, Graham CA, Rainer TH. The normal ranges of cardiovascular parameters in children measured using the Ultrasonic Cardiac Output Monitor. Crit Care Med. 2010;38: 1875–1881. doi:10.1097/CCM.0b013e3181e8adee

[48] Kam P, Power I. Principles of Physiology for the Anaesthetist. CRC Press; 2012. doi:10.1097/00003643-200202000-00021

[49] Mynard JP. Computer modeling and wave intensity analysis of perinatal cardiovacular fucntion and dysfunction. 2011; 76–125.

[50] Heldt T. Computational models of cardiovascular response to orthostatic stress. Massachusetts Institute of Technology. 2004.

[51] Baretta A, Corsini C, Yang W, Vignon-Clementel IE, Marsden AL, Feinstein JA, et al. Virtual surgeries in patients with congenital heart disease: A multi-scale modelling test case. Philos Trans R Soc A Math Phys Eng Sci. 2011;369: 4316–4330. doi:10.1098/rsta.2011.0130

[52] Tanaka T, Arawawa M, Suzuki T, Gotoh M, Miyamoto H, Hirakawa S. Compliance of human pulmonary “venous” system estimated from pulmonary artery wedge pressure tracings - Comparison with pulmonary arterial compliance. Jpn Circ J. 1986;50: 127–139.

[53] Liang F, Senzaki H, Kurishima C, Sughimoto K, Inuzuka R, Liu H. Hemodynamic performance of the fontan circulation compared with a normal biventricular circulation: A computational model study. Am J Physiol - Hear Circ Physiol. 2014;307: H1056–H1072. doi:10.1152/ajpheart.00245.2014
