# Supplementary material for: Ventricular outflow tract obstruction: An in-silico model to relate the obstruction to hemodynamic quantities in cardiac paediatric patients
Source: PLoS One. 2021 Oct 15;16(10):e0258225. doi: 10.1371/journal.pone.0258225 (PMC8519477; doi:10.1371/journal.pone.0258225)
Supplement: S5 File — (DOCX) [file pone.0258225.s005.docx]

**S5 Global sensitivity analysis**

To evaluate the sensitivity of the model, we performed a global sensitivity analysis (SA) which, although not fully exhaustive, gives a useful and meaningful view of the influence of inputs on the outputs. To that aim, we followed the Monte Carlo-based approach proposed by Saltelli [54] and applied to cardiovascular 0D-1D models by Zhang *et al.* [55]. We prescribed an uncertainty of 15% for all the inputs and analysed the behaviour of the sensitivity indices for the primary outputs: the mean aortic pressure ($mP_{ao}$), the mean inferior caval venous pressure ($mP_{ivc}$), the mean pulmonary artery pressure ($mP_{pua}$), and the cardiac output ($CO$). Notice that sensitivity indices that resulted lower than 0.05 were put equal to zero, i.e., the impact of the corresponding parameters on the uncertainties of the model outputs was considered negligible. Fig. S5.1 shows that only few inputs have a detectable influence on the selected outputs. In parti

cular, it can be seen that: i) systemic pressures are mainly influenced by the elastances of the heart; ii) $mP_{pua}$ is affected also by pulmonary vascular resistances; iii) $CO$ is influenced by heart elastances and HR. Moreover, when the inputs vary in the range $\pm$15% the analysed pressures and flow rate exhibit maximum variation around 20% which seems acceptable in the cardiovascular panorama.


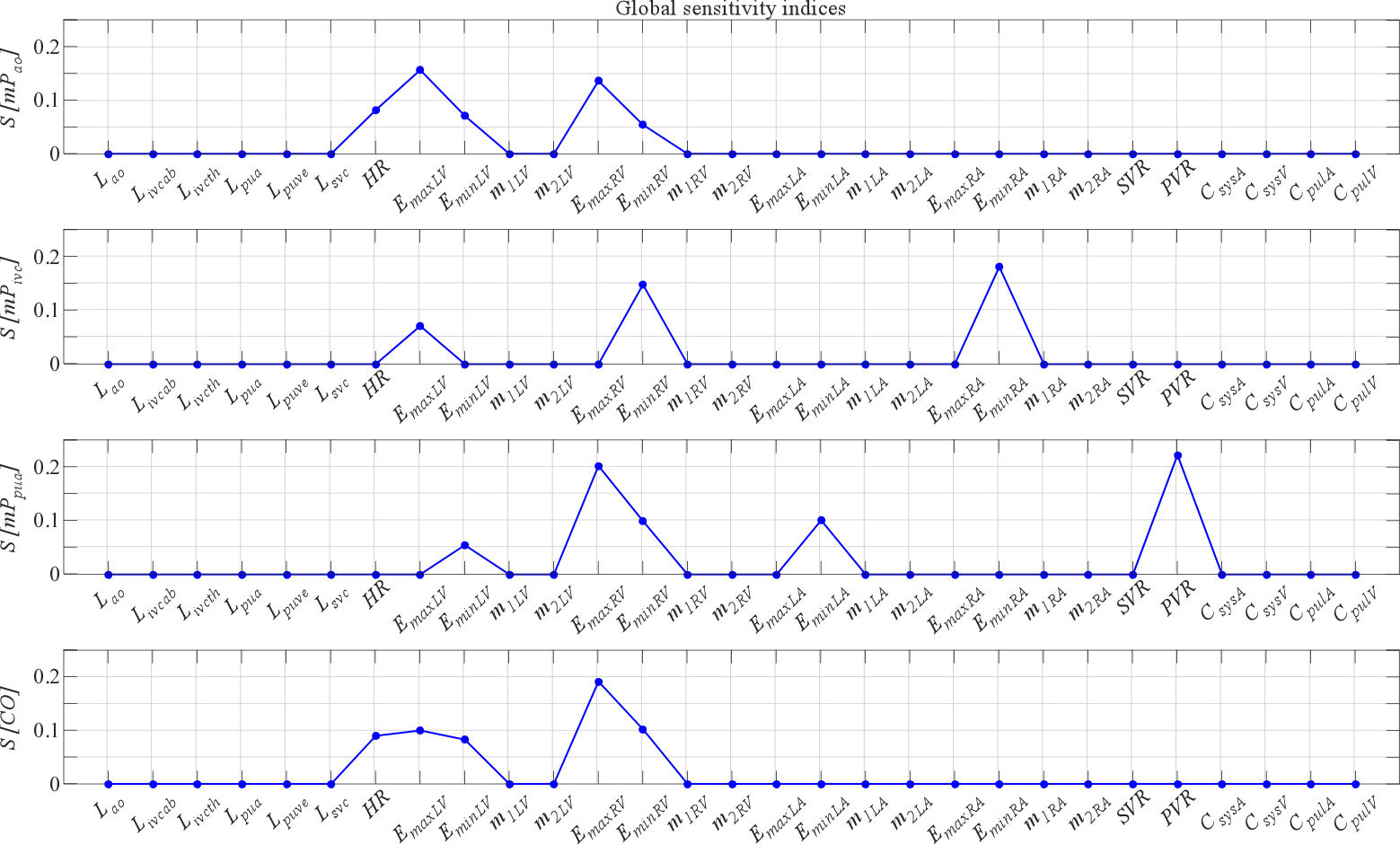


**Fig. S5.1.** Computed global sensitivity indices. $mP_{ao}$, the mean aortic pressure, $mP_{ivc}$, the mean inferior caval venous pressure, $mP_{pua}$, the mean pulmonary artery pressure, and $CO$, the cardiac output. $L_{ao}$, aortic inductance, $L_{ivcab}$, abdominal inferior vena cava inductance, $L_{ivcth}$, thoracic inferior vena cava inductance, $L_{pua}$, pulmonary arterial inductance, $L_{puve}$, pulmonary venous inductance, $L_{svc}$, superior vena cava inductance, HR, heart rate, $E_{{max}_{LV}}$, LV maximum elastance, $E_{{min}_{LV}}$, LV minimum elastance, $m_{1_{LV}}$, LV systolic steepness, $m_{2_{LV}}$, LV diastolic steepness, $E_{{max}_{RV}}$, RV maximum elastance, $E_{{min}_{RV}}$, RV minimum elastance, $m_{1_{RV}}$, RV systolic steepness, $m_{2_{RV}}$, RV diastolic steepness, $E_{{max}_{LA}}$, LA maximum elastance, $E_{{min}_{LA}}$, LA minimum elastance, $m_{1_{LA}}$, LA systolic steepness, $m_{2_{LA}}$, LA diastolic steepness, $E_{{max}_{RA}}$, RA maximum elastance, $E_{{min}_{RA}}$, RA minimum elastance, $m_{1_{RA}}$, RA systolic steepness, $m_{2_{RA}}$, RA diastolic steepness, SVR, systemic vascular resistance, PVR, pulmonary vascular resistance, $C_{{sys}_{A}}$, total systemic arterial compliance, $C_{{sys}_{V}}$, total systemic venous compliance, $C_{{pul}_{A}}$, total pulmonary arterial compliance, $C_{{pul}_{V}}$, total pulmonary venous compliance.

**References**

[54] Saltelli A. Making best use of model evaluations to compute sensitivity indices. Comput Phys Commun. 2002;145: 280–297. doi:10.1016/S0010-4655(02)00280-1

[55] Zhang X, Haneishi H, Liu H. Multiscale modeling of the cardiovascular system for infants, children, and adolescents: Age-related alterations in cardiovascular parameters and hemodynamics. Comput Biol Med. 2019;108: 200–212. doi:10.1016/j.compbiomed.2019.03.021
